# Expansion of a bitter taste receptor family in a polyphagous insect herbivore

**DOI:** 10.1038/srep23666

**Published:** 2016-04-01

**Authors:** Wei Xu, Alexie Papanicolaou, Hui-Jie Zhang, Alisha Anderson

**Affiliations:** 1 CSIRO Food and Nutrition Flagship, Black Mountain, ACT, Australia 2601; 2 School of Veterinary and Life Sciences, Murdoch University, Murdoch, WA, Australia 6150; 3 CSIRO Land and Water Flagship, Black Mountain, ACT, Australia 2601

## Abstract

The Insect taste system plays a central role in feeding behaviours and co-evolution of insect-host interactions. Gustatory receptors form the interface between the insect taste system and the environment. From genome and transcriptome sequencing we identified 197 novel gustatory receptor (GR) genes from the polyphagous pest *Helicoverpa armigera*. These GRs include a significantly expanded bitter receptor family (180 GRs) that could be further divided into three categories based on polypeptide lengths, gene structure and amino acid sequence. Type 1 includes 29 bitter *Gr* genes that possess introns. Type 2 includes 13 long intronless bitter *Gr* genes, while Type 3 comprises 131 short intronless bitter *Gr* genes. Calcium imaging analysis demonstrated that three Type 3 GRs (HarmGR35, HarmGR50 and HarmGR195) can be activated by a crude extract of cotton leaves. HarmGR195, a GR specifically and selectively expressed in adult tarsi, showed a specific response to proline, an amino acid widely present in plant tissues. We hypothesise that the expansion in the *H. armigera* GR family may be functionally tied to its polyphagous behavior. Understanding the molecular basis of polyphagy may provide opportunities for the development of new environmentally friendly pest control strategies.

Chemosensory receptors represent an interface between an insect and its chemical environment, mediating pivotal biological processes such as host finding, mate selection and choice of oviposition sites^1^. One gene family, the gustatory receptors (GRs), plays a central role in co-ordinating insect feeding behaviours^1^. Taste stimuli from the environment are recognized by GRs located on the dendrites of taste sensilla, which are distributed throughout the insect body^1^. Despite a growing body of knowledge about the insect taste system, little is known about the molecular and cellular mechanisms that underlie taste signal recognition or how these signals affect feeding behaviours.

Insect *Gr* genes were first identified from the *Drosophila melanogaster* genome^2^. They have been classified into four clades: CO_2_^3^, GR43a-like^4^, sugar^5^,^6^,^7^,^8^,^9^,^10^ and bitter^11^,^12^. To date, published research on GRs has been focused on *Drosophila*^5^,^6^,^8^,^9^,^10^,^13^,^14^,^15^,^16^,^17^, but with the increasing availability of genomic information from other insect species, such as *Anopheles gambiae*^18^, *Tribolium castaneum*^19^, *Apis mellifera*^20^, and *Acyrthosiphon pisum*^21^, GR research is being extended to a diverse range of species. The recent genome projects of four lepidopteran species, silkworm (*Bombyx mori*)^11^, Monarch butterfly (*Danaus plexippus*)^22^,^23^, Postman butterfly (*Heliconius melpomene*^23^ and Diamondback moth (*Plutella xylostella*)^24^ provide invaluable resources for looking deeply into the lepidopteran gustatory system. 69 GRs were identified from *B. mori*^11^, 58 GRs were identified from *D. plexippu*s^22^,^23^, 73 GRs were identified from *H. melpomene*^23^ and 69 GRs were identified from *P. xylostella*^24^. These four species are all specialist feeders: *B. mori* is a mulberry leave specialist^11^; *D. plexippus* consumes only plants in the milkweed family (Asclepiadacea)^22^; *H. melpomene* feeds on either *Passiflora oerstedii* or *Passiflora menispermifolia*^23^; while *P. xylostella* feeds exclusively on crucifers^24^. Therefore, the study on gustatory systems of these four species can shed light on the interactions between host plants and specialist herbivores but may be less relevant to generalist species like *Helicoverpa armigera*, which include significant pests of human agriculture. *H. armigera* itself is one of the most polyphagous lepidopteran pest species, with larvae that feed on numerous cultivated crops such as cotton, peanuts, soybeans and maize. *H. armigera* would be an ideal species for studying the gustatory receptor repertoire of a polyphagous lepidopteran species. Our hypothesis is that the gustatory system of *H. armigera* may have species-specific features that contribute to its robust polyphagy.

In this study, we utilized genome and transcriptome data from *H. armigera* and manually identified this species repertoire of *Gr* genes. We applied phylogenetic analysis, comparative gene expression, and topological and calcium imaging analyses to structurally and functionally characterize the GR repertoire, in the process uncovering a markedly expanded family of lepidopteran GRs.

## Results

### GR Gene Annotation and Identification

**Figure 1:**
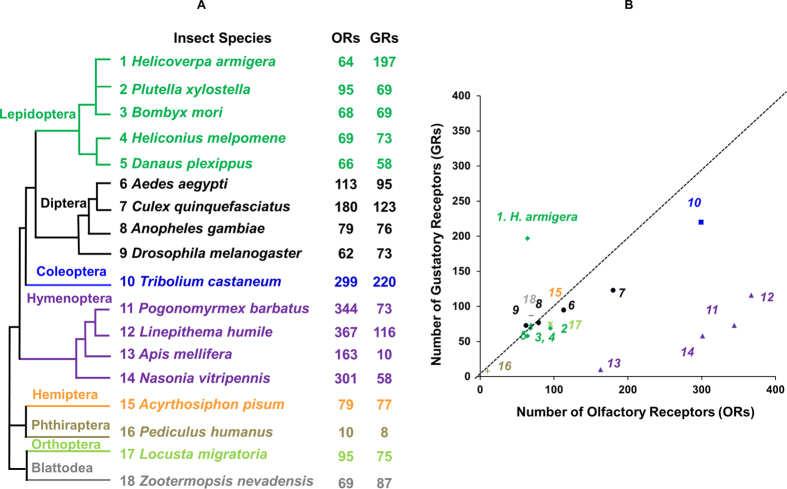
Relationship between numbers of GRs and ORs in different insect species. (**A**) Phylogeny of various insect species and the number of ORs and GRs identified from each species’ genome: 1 *H. armigera*, 2 *P. xylostella*^24^,^28^, 3 *B. mori*^4^,^11^, 4 *H. melpomene*^23^ and 5 *D. plexippus*^22^,^23^ (green); 6 *A. aegypti*^48^, 7 *C. quinquefasciatus*^49^, 8 *A. gambiae*^18^ and 9 *D. melanogaster*^2^, (black); 10 *T. castaneum* (blue)^19^; 11 *P. barbatus*^50^, 12 *L. humile*^51^, 13 *A. mellifera*^20^, and 14 *N. vitripennis*^52^, (purple); 15 *A. pisum* (orange)^21^; 16 *P. humanus*^53^ (brown), 17 *L. migratoria*^54^ (light green) and 18 *Z. nevadensis*^55^ (grey). (**B**) A map of numbers of ORs (Y-axis) and GRs (X-axis) of insect species mentioned above. Lepidoptra (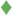), Diptera (●), Hymenoptera (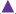), Coleoptera (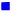), Hemiptera (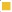), Phthiraptera (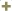), Orthoptera (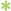) and Blattodea (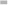).

A bioinformatics screen of the *H. armigera* genome and transcriptome data revealed a total of 197 GR and 64 odorant receptor (OR) genes (Fig. 1A). All identified *H. armigera* GR nucleotide and amino acid sequences, distributions on scaffolds and other information are available online (Table S1). In comparison with all insect species with currently available genomes, *H. armigera* showed the second highest number of GRs, 197. This is the highest number of GRs identified in a lepidopteran genome with only the red flour beetle *T. castaneum* having more, 220 (Fig. 1A). Most insect species studied possess comparable total numbers of ORs and GRs (Fig. 1B). *H. armigera* is unique among insects in having an increase in total GR numbers relative to OR numbers (Fig. 1B). Of the exceptions, the honeybee, *Apis mellifera* has an expansion of ORs (163) but possess only 10 GRs^20^ (Fig. 1A) and wasps and ants also have many more ORs than GRs in their genomes (Fig. 1A).

### Phylogenetic analysis

**Figure 2:**
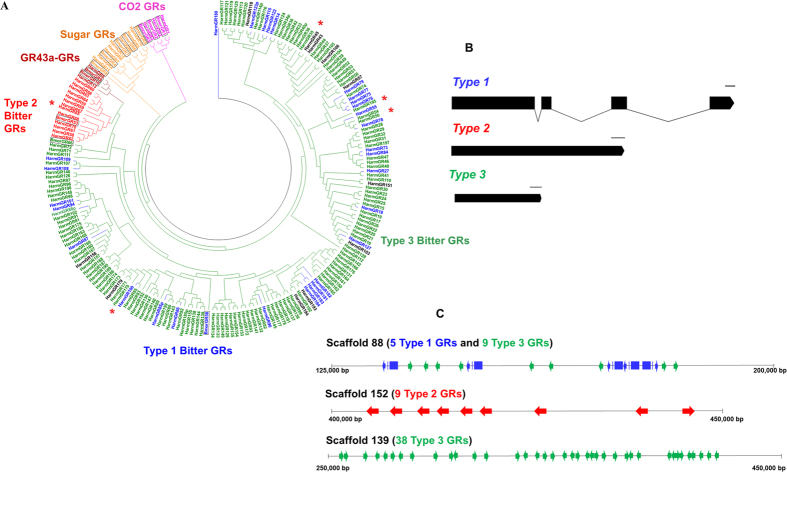
Phylogenetic analysis and characterisation of *H. armigera* GRs. (**A**) Phylogenetic analysis of selected *B. mori* and all *H. armigera* GRs. CO_2_ GRs (Purple), Sugar GRs (Orange), GR43a-like GRs (dark red) are labelled. For bitter GRs, Type 1 (blue), Type 2 (red) and Type 3 (green) are also labelled. Unknown types (partial sequences and pseudogenes) are labelled in black. Type 2 GRs (HarmGR65) and Type 3 GRs (HarmGR35, 50, 170 and 195) which were used in topological and functional characterization experiments are asterixed. *B. mori* GRs are framed. (**B**) The gene structures of Type 1, Type 2 and Type 3 bitter GRs in lepidopteran species. Bars represent exon sequence, lines represent intron sequence. (**C**) Examples of the distribution and organization of some bitter GRs in *H. armigera* genome scaffolds.

To investigate the types of GRs that were expanded, we selected thirteen GRs from *B. mori* as representative of the four insect GR subfamilies and compared them in a phylogenetic analysis to all 197 GRs from *H. armigera* (Fig. 2A). A phylogeny tree with all *B. mori* GRs and *H. armigera* GRs was provided (S7 Figure). The analysis reveals HarmGR1–3 are members of the CO_2_ receptor subfamily^25^ (Fig. 2A). HarmGR4–8 and 10–12 are members of the insect sugar receptor subfamily and share 46–99% identity with other known moth sugar GRs (Table S1). HarmGR9 and 13 are members of the GR43a-like receptor subfamily^26^. The remaining 184 HarmGRs form a large “bitter” receptor subfamily with the representative *B. mori* bitter GRs (BmorGR53, 58 and 66) (Fig. 2A). Four of these GRs, (HarmGR57, 158, 179 and 193) contain a stop codon in the ORF and are likely to be pseudogenes. Thirteen of these GRs (HarmGR44, 45, 63, 78, 85, 88, 103, 106, 118, 123, 151, 185 and 194) are partial sequences, missing their N- or C-terminus.

We then compared the total numbers of CO_2_, sugar, GR43a-like and bitter GRs found in the *H. armigera* genome with those of other Lepidoptera; *B. mori*, *D. plexippus*, *H. melpomene* and *P. xylostella* (Table 1). The largest difference is in the bitter receptor family, comprising 180 GRs in *H. armigera*, which is three times the number found in *B. mori* (51 GRs), *H. melpomene* (57 GRs), or *P. xylostella* (49 GRs) and four times more than in *D. plexippus* (40 GRs)^4^,^11^,^22^,^23^,^27^,^28^. There are also small differences in the total numbers of genes for the remaining GR sub-families. For the CO_2_ GRs, *P. xylostella* has five while the other four species possess three (Table 1). The number of sugar GRs varies from four to nine across the five species. There are more GR43a-like GRs in the two butterflies (5 or 6 GRs) and *P. xylostella* (7 GRs) than in *B. mori* and *H. armigera* (2 GRs) (Table 1).

### Bitter receptors

To better understand the bitter receptor expansion in *H. armigera* we performed an in-depth phylogenetic analysis of the subfamily (Fig. 2A). The three *B. mori* bitter GRs (BmorGR53, 58 and 66) selected for the phylogenetic comparison with *H. armigera*, represent three different structures of bitter receptors. BmorGR58 contains four exons and three introns and is representative of the majority of other *B. mori* bitter GRs (Fig. 2A). BmorGR53 is an intronless bitter GR, the only of its kind found in *B. mori*, while BmorGR66 is a shorter intronless bitter GR^4^. We were able to group most lepidopteran full-length bitter GRs into three categories based on their lengths and sequences (Fig. 2B and Table 1). Type 1 describes *Gr* genes, like BmorGR58, that encode approximately 400 amino acids (Fig. S5) and contain 3~4 exons (Fig. 2B). Type 2 comprises a group of long intronless GRs, also encoding approximately 400 amino acids (Fig. S5), like BmorGR53^23^,^27^. Type 3 comprises short intronless genes (Fig. 2B and Table 1) encoding 200~350 amino acids (Fig. S5), like BmorGR66^4^. Compared with other lepidopteran species, *H. armigera* has a low total number of Type 1 GRs but a larger total number of both Type 2 and especially Type 3 bitter GRs (Table 1). Phylogenetically, the *H. armigera* Type 2 *Gr* genes cluster together to form a monophyletic branch, while Type 1 and Type 3 are interspersed in the bitter GR sub-family (Fig. 2A). Analysis of their genomic distribution revealed Type 2 or Type 3 GRs are often clustered together (Fig. 2C). For example, 38 Type 3 *Gr* genes are localized in a tandem array within 0.2 megabases on scaffold 139 (Fig. 2C) and nine Type 2 genes are clustered within 0.05 megabases on scaffold 152 (unpublished data). Five Type 1 and nine Type 3 *Gr* genes are interspersed with each other on scaffold 88 (Fig. 2C).

**Figure 3:**
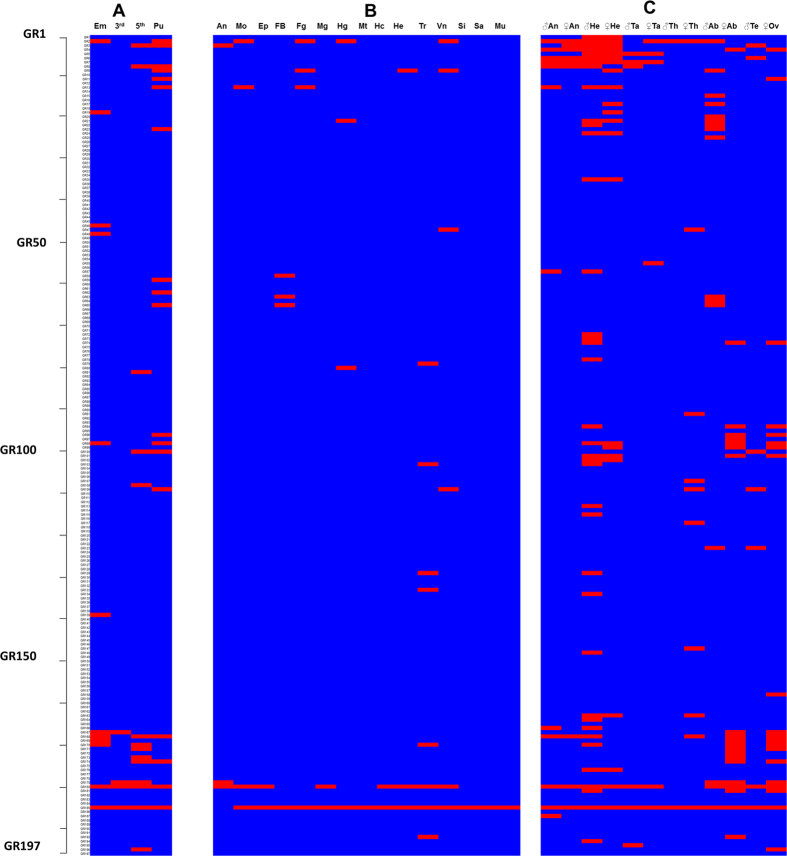
*H. armigera* GR expression as revealed by read mapping. Red box indicates the presence of reads uniquely mapping to the coding region of each receptor gene model while blue box indicates no expression detected. The expression of GRs in various tissues include: (**A**) embryos (Em), 3^rd^-instar larvae whole bodies (3^rd^), 5^th^-instar larvae whole bodies (5^th^) and pupae (Pu); (**B**) larvae antennae (An), mouthparts (Mo), epidermis (Ep), fat body (FB), foreguts (Fg), midgets (Mg), hindguts (Hg), malpigian tubules (Mp), hemocytes (Hc), hearts (He), trachea (Tr), ventral nerve (NV), silk glands (Si), salivary glands (Sa) and muscle (Mu) of 5^th^ instar; (**C**) male antennae (♂An), female antennae (♀An), male heads with antennae (♂He), female heads with antennae (♀He), male tarsi (♂Ta), female tarsi (♀Ta), male thorax (♂Th), female thorax (♀Th), male abdomens (♂Ab), female abdomens (♀Ab), male testes (♂Te) and female ovaries (♀Ov) from day 0 to day 5 adults.

As Type 3 *Gr* genes are short intronless genes, we wanted to verify if these were complete genes and not artefacts of incorrect genome assembly. We performed RT-PCR on 20 of these genes to verify their sequence and expression and show all 20 are as predicted from the genome (data not shown). Within our transcriptome libraries (established from 31 RNA sequencing libraries based on different tissues and life stages, unpublished data), we identified the complete ORF sequences for seven Type 3 *Gr* genes (HarmGR24, 35, 50, 168, 169, 171 and 174) confirming the genomic sequences are complete. We also used 3′RACE to sequence the ORFs of two Type 3 genes (HarmGR17 and HarmGR19) and confirm the positions of their stop codons are the same as those identified from the genome (S4 Data). Both HarmGR17 and HarmGR19 are highly expressed in female adult heads (Fig. 3). We did not perform 5′ RACE due to the high level of conservation at the 5′ terminal. We also confirmed that the cDNA sequences of the genes used in our functional studies, HarmGR35, 50, 65, 170 and 195 (Fig. S3), matched the sequences annotated from the genome.

### GR gene expression profile

To help characterise the potential function of *H. armigera* bitter receptor candidates, especially members of the expanded Type 3, we built expression profiles for them from 31 transcriptomic libraries (unpublished data) (Fig. 3). We detected the presence of 84 of the total repertoire of 197 GRs. We used a conservative ‘cutoff’ value to judge if a GR is detected or not in the transcriptome libraries, which is based on relative abundance^29^. As GRs are lowly expressed, it is difficult to assess if non-detection means there is no expression in a tissue or if the level of expression is very low but would result in expression of functional proteins. As there are no biological repeats of the transcriptomic libraries we cannot meaningfully compare expression levels of *Gr* genes between the different libraries. We can, however, report the number of GRs expressed within these different libraries (Fig. 3A). Across the different developmental stages, four GRs were detected from 3^rd^ instar larvae, twelve were detected from embryos and 5^th^ instar respectively, and eighteen were detected from pupae (Fig. 3A). Across the libraries of 15 different larval tissues (Fig. 3B) and 13 adult tissues (Fig. 3C) we found twenty *Gr* genes were expressed in larval tissues (Fig. 3B) and 73 *Gr* genes was detected in adult tissues (Fig. 3C). Most *HarmGr* genes detected in adults were found in the heads, abdomens or female ovaries (Fig. 3C). Some individual *Gr* genes were expressed in multiple tissues. For example, HarmGR185 was expressed in nearly all the tested samples except larval antennae (Fig. 3A). HarmGR180 is detected in 22 different tissues. Conversely some individual *Gr* genes were only detected in specific tissue(s). For example, HarmGR35 was only detected in the adult heads (Fig. 3C), HarmGR65 was detected in only two tissues, the larva fat body (Fig. 3B) and male adult abdomen (Fig. 3C) and HarmGR195 was only detected in adult tarsi (Fig. 3C). Given the low number of cells expressing GRs and the expected low expression levels, it is probable that their expression is not always detectable by transcriptome sequencing.

### Topology

**Figure 4:**
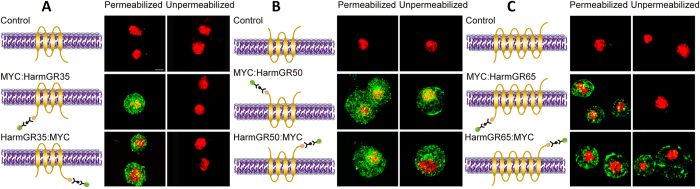
Topological studies of *H. armigera* “bitter” receptors. Immunohistochemical localisation of the N-termini and the C-termini of (**A**) HarmGR35, (**B**) HarmGR50 and (**C**) HarmGR65. HarmGRs were expressed in native form or fused with two myc epitopes at its N or C-termini. Cells were labelled with mouse anti-myc and Alexa-labelled anti-mouse antibodies to determine the accessibility of a myc antigen under permeabilised or unpermeabilised conditions. Schematic of expression constructs for untagged Grs (controls), N-terminally MYC-tagged GRs (MYC:GRs) and C-terminally MYC-tagged Grs (GRs:MYC). Note: The actual number of transmembrane domains present is not known. Green indicates MYC-directed Alexa immunofluorescence. Red indicates DAPI nuclear counter stain. Scale Bar = 5 μm.

Insect GRs have been shown to have a similar topologies to insect ORs, with seven transmembrane domains, an intracellular N-term and extracellular C-term^27^. As Type 3 receptors are shorter in length and likely to have fewer transmembrane domains than the longer GRs, we analysed their topology. Using the algorithms TMPred, HMMTOP and TMHMM (Fig. S6) we predict that the *H. armigera* Type 3 bitter receptors have between three and seven transmembrane domains (TMDs. Type 1 and Type 2 GRs are predicted to have five to nine TMDs (Fig. S6). We selected two Type 3 receptors (GR35 and 50) expressed in the male adult head and one Type 2 receptor (GR65) expressed in the fat body, and expressed them as N- and C-terminally MYC-tagged fusions in S2 cells. Untagged receptors were used as controls. In all three cases, strong green immunofluorescence could be visualized from permeabilized cells transfected with either MYC: HarmGR or HarmGR: MYC (Fig. 4). In contrast, when cells were not permeabilised, we observed three different results. No fluorescence was observed on either N- or C-terminally tagged HarmGR35, indicating the tags are intracellular (Fig. 4A). Green fluorescence was seen in cells transfected with both N- and C- terminally tagged HarmGR50, indicating both tags are extracellular (Fig. 4B). Green fluorescence was seen for only C- terminally tagged HarmGR65, HarmGR65: MYC, but not from the N-terminally tagged HarmGR65 (Fig. 4C). These results indicate that HarmGR35, HarmGR50 and HarmGR65 exhibit three different topological structures. The N and C-termini of HarmGR35 are both intra-cellular. The N and C-termini of HarmGR50 are both extra-cellular. HarmGR65, a member of the Type 2 bitter receptor family, predicted to have seven TMDs, has the same topology as BmorGR53^27^ with an intracellular N-terminus and an extracellular C-terminus.

### Functional characterization

**Figure 5:**
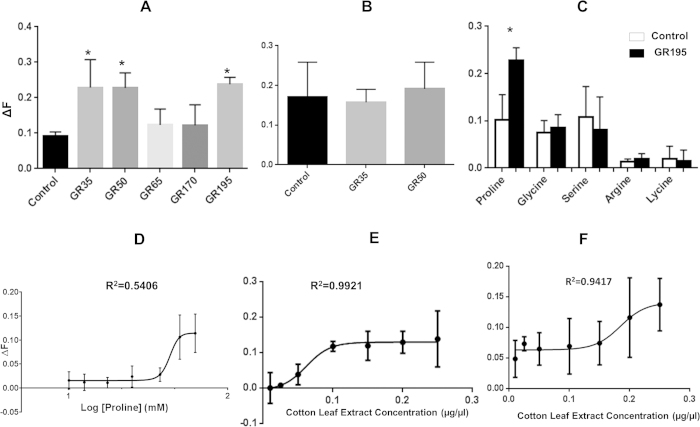
Functional characterization of GR35, GR50 and GR195 with leaf extracts and amino acids. (**A**) Calcium imaging analysis of HarmGR35, HarmGR50, HarmGR65, HarmGR170 and HarmGR195 exposed to a crude extract of cotton leaves. (**B**) Calcium imaging analysis of HarmGR35 and HarmGR50 in response to crude extract from tobacco leaves. (**C**) HarmGR195 in response to 50 mM amino acids; proline, glysine, serine, arginine and lysine. (**D**) Concentration-dependent response curve for HarmGR195 to proline. (**E**) Concentration-dependent response curves for HarmGR35 and (**F**) HarmGR50 to crude extract from cotton leave. The average responses of control cells have been subtracted. Error bars indicate the calculated standard divisions of the difference. Analysis of the statistical significance between each response and control was conducted by Dunnett tests as part of one-way ANOVA using arcsine transformation. *p < 0.05.

Characterising the function of the entire Type 3 GR category would be extremely challenging due to the labour intensive methods needed for functional studies and the lack of information on possible ligands. We therefore focused on a small number of Type 3 *Gr* genes to establish whether Type 3 GRs exhibited responses to physiologically relevant ligands. We chose genes expressed in adult tissues relevant to the insect taste system. HarmGR35 and HarmGR50 are expressed in adult male heads (Fig. S3); HarmGR195 is expressed in adult tarsi (Fig. 3) and HarmGR170 is expressed in the adult female abdomen (Fig. S3). We also chose a Type 2 GR (HarmGR65), which is expressed in only 2 libraries, the larval fat body and the male abdomen. Using quantitative calcium imaging, we tested whether, when expressed in Sf9 cells, the receptors would respond to compounds present in crude extracts of host plants. Sf9 cells transfected with an empty expression vector (negative control) showed a low but detectable response to the extract, possibly due to membrane receptors that are expressed natively in the cells. A crude extract of cotton leaves initiated significantly higher responses from cells transfected with HarmGR35, HarmGR50 or HarmGR195, when compared to the negative control, HarmGR65 and HarmGR170 (Fig. 5A). These responses were dose-dependent (Fig. 5E,F) with EC_50_ values of 0.062 ± 0.006 (SEM, N = 3) extract μg/μL for HarmGR35 and 0.185 ± 0.017 (SEM, N = 3) extract μg/μL for HarmGR50.

For HarmGR35 and 50, we also tested a crude extract of tobacco leaves, since tobacco is also a host for *H. armigera*. However, the crude extract of tobacco leaves did not trigger significant responses from either HarmGR35 or HarmGR50 (Fig. 5B).

We further tested the HarmGR195 response to the amino acids, proline, glycine, serine, arginine and lysine, as this GR is expressed in adult tarsi (Fig. 3 and Fig. S3), which have been shown by electrophysiological studies to detect amino acids^30^. We found HarmGR195 is selectively activated by proline (ΔF = 0.106, *p* < 0.05) but not glycine, serine, arginine or lysine at 50 mM (Fig. 5C). The response to proline is dose-dependent with an EC_50_ = 43 ± 7 mM (SEM, N = 3) (Fig. 5D).

## Discussion

After manual curation of the genome and analysis of *H. armigera* taste receptor genes, we found an expanded subfamily of gustatory receptors in *H. armigera* compared with other Lepidoptera. The total number of GRs and ORs may be linked to an insect species’ behaviour and ecology. For example, the honeybee, *A. mellifera* has an expansion of the olfactory receptor gene family (163 ORs)^20^ compared with other insects, presumably enhancing its olfactory ability and therefore facilitating the typical foraging and social behaviour of bees^20^. On the other hand, the honey bee genome contains only 10 *Grs*^20^. It has been hypothesized that bees have limited need for *Grs* for plant secondary metabolite discrimination since flowering plants have evolved visual and olfactory cues to attract bees^20^. We postulate that the expansion of *H. armigera* GRs may be linked to this species’ capacity for being a successful generalist as the expansion presumably broadens the range of plant secondary metabolites detected by this species.

We focused our comparison of the GRs on lepidopteran species because of the high degree of amino acid dissimilarity in GRs across even modest evolutionary distances. In comparison with *B. mori*’s 69 GRs, *D. plexippus*’s 58 GRs, *H. melpomene*’s 73 GRs and *P. xylostella*’s 69 GRs, the nearly three fold expansion of the GR repertoire in *H. armigera* is mainly in the bitter receptor family. Lepidopteran gustatory receptors have been classified into ‘CO_2_’, ‘GR43a-like’, ‘sugar’ and ‘bitter’ clades^28^. While CO_2_ and ‘sugar’ receptors have been confirmed to respond to CO_2_^25^, sugars^27^ and ‘GR43a-like’ have been confirmed to also respond to sugars^26^, there are no studies confirming that ‘bitter’ receptors respond to bitter tastants. Here we named this large clade of GRs as putative “bitter receptors” because they are grouped in the same branch with *B. mori* “bitter receptors”^11^ in the phylogenetic analysis, whose ligands are not yet identified (Fig. S7). From the expression profile on 31 libraries, only half of the total GRs were detected above the conservative cut-off value. *Gr* gene expression itself is very low and may only be expressed in very few cells within a given tissue hence our conservative cut off may exclude some GRs that are actually functional in the tissue. For example, GR50 is not detected in male heads in our expression profile but can be detected using RT-PCR. When we went back to the raw data we did detect GR50 reads but at a very low level. A further reason for low expression levels in this study may be because lab colonised *H. armigera* were used in library construction. This colony has been fed on artificial diet for over 10 years which may have resulted in lower GR expression compared with wild insects.

Some GRs are detected in internal tissues like gut, heart and fat body suggesting they may be functioning in internal nutrient detection, as reported in *Drosophila*^31^. *H. armigera* GRs were also detected in embryo stages, suggesting they play a role in insect development as previously reported in *Drosophila*^32^.

We further categorised the bitter GRs into three ‘types’ based on gene structure and length. Type 1 exhibit structural features which are conserved across lepidopteran species GRs while Type 2 and Type 3 are intronless GRs that are less commonly found in the available genomes of other Lepidoptera. Type 2 refers to those genes that are relatively long (>400 AA) and Type 3 as those that are relatively short (<360 AA) (Fig. 2). Interestingly, in mammals, intronless genes are shown to be more lowly expressed, present in a narrower range of tissues and evolving faster than intron containing genes^33^. The majority of the bitter receptors in the *H. armigera* expansion fall into the Type 3 category.

Type 3 *Gr* genes are often arranged in the *H. armigera* genome in gene clusters (Fig. 2), suggesting they arise from a few ancestral genes that have undergone successive duplications. Previous to this study only a limited number of lineage specific expansions in ORs or GRs have been observed in Lepidoptera^28^. It is also suggested that the common ancestor of the Lepidoptera had a very small number of *Gr* genes, particularly within the bitter receptor clade, so further analysis of the genomic structure of these clusters in *H. armigera* may shed light on the evolution of this unique GR expansion.

It would be extremely challenging to comprehensively de-orphan all of these Type 3 GRs, instead we focused on testing whether at least some Type 3 GRs exhibited responses to physiologically relevant ligands. Since there is little behavioural or neurophysiological information on ligands that could activate these receptors, we used crude extractions of leaves from cotton and tobacco, two common host plants for *H. armigera* that are known to deploy secondary metabolites as defence against herbivores^34^. Of the five genes we tested, HarmGR35, HarmGR50 (expressed in adult head) and HarmGR195 (expressed in adult tarsi) showed responses to a crude extract of cotton leaves. It is interesting that GR35 and 50, expressed specifically in male head, detect plant compounds. Males also use plant compounds to find food sources and might use host-plant chemical cues to identify the habitat of calling females^35^.

HarmGR195, which is specifically expressed in the adult tarsi, responded to proline in a dose-dependent manner. Plant nectars may contain up to 2 mM proline^36^, and this high level of proline is thought to be an attractant, as several species of insects prefer high-proline nectars^36^. Given that it is highly expressed on the tarsi, HarmGR195 may have a role in regulating the insects’ feeding or oviposition.

Insect GRs are predicted to have seven TMDs, as is typical for insect odorant receptors^2^,^20^,^37^,^38^ and the topologies of insect GRs are predicted or confirmed to have an intracellular N-terminus and an extracellular C-terminus^11^,^27^,^37^. We found two different topologies for the two Type 3 GRs; HarmGR35, has two intracellular termini, while HarmGR50, has two extracellular termini, indicating both have an even number of TMDs. Type 3 GRs are also predicted to have fewer TMDs, making them the first report of an insect GR family with multiple topologies (Fig. S6).

Previous studies on *Drosophila* GRs have shown that insect GRs form functional heteromultimers *in vivo*^3^,^9^,^12^ with genetic studies indicating that co-expression of multiple GRs is essential for the detection of compounds like CO_2_, sucrose, D-glucose and trehalose^3^,^6^,^8^,^9^,^39^. *In vitro* studies with GRs from *B. mori*, BmorGR8 and BmorGR9 have shown that responses to *myo*-inositol or D-fructose do not require the coexpression of other GRs^4^,^27^, however these studies do not conclude that GRs can function as homo-oligomers because the lepidopteran-derived Sf9 cells used may express native co-receptors. For example, Orco, the canonical OR co-receptor is expressed in Sf9 cells and probably enables the correct functioning of odorant receptors in Sf9 heterologous assays^40^. In this study Type 3 receptors were able to function alone in Sf9 cells, however it is possible that multiple GRs are required for function *in vivo*.

In summary, we have discovered *H. armigera* has evolved a high number of *Gr* genes, most of which are intronless and belong to the bitter gustatory receptor clade. We have shown these genes have topologies not previously seen in insect GRs and at least three of them show functional responses that could be related to their feeding behaviour. We propose this expansion in bitter receptors allows *H. armigera* to detect a broad range of plant secondary metabolites and contributes to the highly successful polyphagous behaviour of this species.

## Materials and Methods

### Insects and cell culture

*H. armigera* (CSIRO general laboratory (GR) rearing strain) were fed an artificial diet as previously described^41^. *Spodoptera frugiperda* Sf9 and *D. melanogaster* Schneider S2 cells were cultured at 28 °C as previously described^26^.

### Gene annotation and analysis

Genes encoding GRs were identified from the contigs of the *H. armigera* genome and transcriptome assemblies, using tBLASTn searches with known *D. melanogaster* and *B. mori* GR sequences (Table S1). Amino acid sequences were used for phylogenetic analysis in MEGA 5.1^42^. A maximum-likelihood tree was calculated using default settings and the Jones-Taylor-Thornton (JTT) model with partial deletions and 1000 bootstrap replications. TMpred (http://www.ch.embnet.org/software/TMPRED_form.html)^43^, HMMTOP (http://www.enzim.hu/hmmtop/)^44^ and TMHMM (http://www.cbs.dtu.dk/services/TMHMM/)^45^ were used to predict transmembrane domains.

### Gene expression profile

RNAseq libraries were prepared by the *Helicoverpa* genome consortium in the following tissues: antennae, mouthparts, epidermis, fat body, foregut, midgut, hindgut, malpighian tubules, haemocytes, hearts, trachea, ventral nerve, silk glands, salivary glands and muscle of 5^th^ instars; male antennae, female antennae, male head, female head, male tarsi, female tarsi, male thorax, female thorax, male abdomens, female abdomens, male testes and female ovaries from day 0 to day 5 adults; embryos; and whole bodies of 3^rd^-instars, 5^th^-instars and pupae. RNAseq data was pre-processed using the default settings of Just_Preprocess_My_Reads (http://justpreprocessmyreads.sourceforge.net), which conducts mild quality control and trimming, pooled and assembled using Trinity-RNASeq, using the default settings^46^. Open reading frames (ORFs) were predicted using the TranscriptDecoder software in Trinity-RNASeq (http://sourceforge.net/projects/transdecoder) with the PFAM option. *In silico* expression profiles were generated using DEW (http://dew.sourceforge.net/), as described^29^.

### Molecular biology

cDNA samples were synthesized and RT-PCR was performed as previously described^29^. 3′ RACE PCR was performed using a SMART RACE cDNA amplification kit with universal and gene-specific primers (Table S2) according to the manufactures’ manuals. PCR products were purified using QIAquick gel extraction reagents (Qiagen, USA), cloned into the pGEM-T Easy vector (Promega, USA) and subsequently sequenced. Successfully cloned full-length ORF sequences were further amplified using specific primers (Table S2) and cloned into the pIB/V5-His vector for expression in insect Sf9 or S2 cells, followed by immunocytochemical studies and calcium imaging analysis.

### Immunocytochemistry

S2 cells were subcultured on poly-L-Lysine coated coverslips in 6-well plates and transfected with 1 μg plasmid [PIB/V5-His vector plus a *H. armigera* GR (HarmGR) as control, or MYC-epitope tagged HarmGR] with 6 μL of Fugene HD transfection reagent (Promega, USA) in 200 μL of medium per well. Forty-eight hours after transfection, immunofluorescence analysis was performed under permeabilised and non-permeabilised conditions as previously described^26^.

### Calcium Imaging

Sf9 cells were plated into 12-well plates and left to settle for 20 min before being transfected by 500 ng of plasmid (pIB/V5-His vector as control or pIB/V5-His-HarmGR vector) and 3 μL of Fugene HD transfection reagent (Promega, USA) in 100 μL per well of Sf-900 medium (Invitrogen, USA). Forty-eight hours after transfection, cells were prepared for calcium imaging and data analysis as described previously^26^,^40^,^47^. Graphpad Prism5 and Microsoft Excel 2012 were utilized for data analysis.

### Tastants

One gram of fresh cotton (*Gossypium hirsutum*) or tobacco (*Nicotiana tabacum*) leaves were ground in 4 mL 1× Hank’s balanced salt solution (HBSS) buffer and centrifuged to extract soluble compounds (0.25 μg/μL), which were used directly (or diluted) to characterized selected HarmGRs using calcium imaging analysis. Proline (purity ≥99%) was purchased from Sigma-Aldrich.

## Additional Information

**How to cite this article**: Xu, W. *et al.* Expansion of a bitter taste receptor family in a polyphagous insect herbivore. *Sci. Rep.* **6**, 23666; doi: 10.1038/srep23666 (2016).

## Supplementary Material

Supplementary Table

Supplementary Information

## Figures and Tables

**Table 1:** Numbers of CO_2_, sugar, GR43a-like and bitter GRs (Type 1, Type 2 and Type 3) in lepdiopteran species *H. armigera*, *B. mori*, *D. plexippus*, *Plutella xylostella* and *H. melpomene*.

Insect Species	CO2GRs	SugarGRs	GR43a-like	Pseudo	Bitter GRs				
					Total	T1	T2	T3	Unkwn
***D. plexippus***	3	9	5	1	40	26	1	1	12
***H. melpomene***	3	7	6	0	57	50	6	1	0
***B. mori***	3	4	2	9	51	47	1	1	2
***P. xylostella***	5	5	6	4	49	21	12	0	16
***H. armigera***	3	8	2	4	180	31	13	129	7

There is a large expansion in the number of bitter GRs of *H. armigera*. The expansion is mostly in the Type 3 bitter GR family.
