# Supplementary material for: Expansion of a bitter taste receptor family in a polyphagous insect herbivore
Source: Sci Rep. 2016 Apr 1;6:23666. doi: 10.1038/srep23666 (PMC4817054; doi:10.1038/srep23666)
Supplement: Supplementary Information [file srep23666-s2.pdf]

Expansion of a bitter taste receptor family in a polyphagous insect herbivore

Wei Xu[a,c], Alexie Papanicolaou [b,d], Hui-Jie Zhang[a] and Alisha Anderson[a]

S2 Primer sequences for the RT-PCR, 3'RACE, calcium imaging and topology analysis.

Purpose	Primer Name	Primer Sequences (5' to 3')
RT-PCR	*Actin-f*	GGGTTCGCGGGCGACGAC
	Acting-r	CGCCACTCGCAGCTCGTTGTAGAAG
	HarmGR35-f	ATGGTCATGATGATCGATTCTTTAGTCAG
	HarmGR35-r	CTACCTAAAAAATACTACCATTAATTTG
	HarmGR50-f	ATGACATTGAATATATTGAATGTG
	HarmGR50-r	TCAATAACTCACCGTAATCTGGAGGTGC
	HarmGR65-f	ATGGAGTTCGGAAAAGATCAGGACAATG
	HarmGR65-r	TTAGATAAAGTGAGATATTTGTATAAG
	HarmGR170-f	ATGTGTTCTTATTTCAAAGAATATTTTG
	HarmGR170-r	TTAACAAGTACTATCAAACCATACCATG
	HarmGR195-f	ATGGAAATTAAATCGTCCAG
	HarmGR195-r	TTATTGATGTAGGTACAGCTTAC

Calcium Imaging	*HarmGR35-F*	GCAAGCTTATGGTCATGATGATCGATTCTTTAGTCAGC
	HarmGR35-R	GCTCTAGACTACCTAAAAAATACTACCATTAATTTGACAG
	HarmGR50-F	GCAAGCTTATGACATTGAATATATTGAATGTGTATT
	HarmGR50-R	GCTCTAGATCAATAACTCACCGTAATCTGGAGGTGCGAG
	HarmGR65-F	GCAAGCTTATG GAGTTCGGAAAAGATCAGGACAATG
	HarmGR65-R	GCTCTAGATTAGATAAAGTGAGATATTTGTATAAG
	HarmGR170-F	GCGGATCCATGTGTTCTTATTTCAAAGAATATTTTG
	HarmGR170-R	GCTCTAGA TTAACAAGTACTATCAAACCATACCATG
	HarmGR195-F	GCGGATCCATGGAAATTAAATCGTCCAG
	HarmGR195-R	GCTCTAGA TTATTGATGTAGGTACAGCTTAC

3'RACE	*HarmGR17-3RACE1*	GTTGTCTCCATACTTATAAGAGTTTGTTG
	HarmGR17-3RACE2	GCGCTTTTACACTACCGGATTGTACGGTC
	HarmGR19-3RACE1	CCATACTCCGCAAACGCCTCGAGCAAGATC
	HarmGR19-3RACE2	GTCTTCTTATTTAAATACAAATCACATTG

Topology	*MYCHarmGR35-F*	GCAAGCTTATG**GAGCAGAAGTTGATTTCAGAGGAGGACTTGGAGCAGAA GTTGATTTCAGAGGAGGACTTG**GTCATGATGATCGATTCTTTAGTCAGCAG C
	MYCHarmGR35-R	GCTCTAGACTACCTAAAAAATACTACCATTAATTTGACAG
	HarmGR35MYC-F	GCAAGCTTATGGTCATGATGATCGATTCTTTAGTCAGC
	HarmGR35MYC-R	GCTCTAGACTA**CAAGTCCTCCTCTGAAATCAACTTCTGCTCCAAGTCCTC CTCTGAAATCAACTTCTGCTC**CCTAAAAAATACTACCATTAATTTGACAG
	MYCHarmGR50-F	GCAAGCTTATG**GAGCAGAAGTTGATTTCAGAGGAGGACTTGGAGCAGAA GTTGATTTCAGAGGAGGACTTG**ACATTGAATATATTGAATGTGTATT
	MYCHarmGR50-R	GCTCTAGATCAATAACTCACCGTAATCTGGAGGTGCGAG
	HarmGR50MYC-F	GCAAGCTTATGACATTGAATATATTGAATGTGTATT
	HarmGR50MYC-R	GCTCTAGATCA**CAAGTCCTCCTCTGAAATCAACTTCTGCTCCAAGTCCTC CTCTGAAATCAACTTCTGCTCATAACTCACCGTAATCTGGAGGTGCGAG**
	MYCHarmGR65-F	GCAAGCTTATG**GAGCAGAAGTTGATTTCAGAGGAGGACTTGGAGCAGAA GTTGATTTCAGAGGAGGACTTG**GAGTTCGGAAAAGATCAGGACAATG
	MYCHarmGR65-R	GCTCTAGATTAGATAAAGTGAGATATTTGTATAAG
	HarmGR65MYC-F	GCAAGCTTATG GAGTTCGGAAAAGATCAGGACAATG
	HarmGR65MYC-R	GCTCTAGATTA**CAAGTCCTCCTCTGAAATCAACTTCTGCTCCAAGTCCTCC TCTGAAATCAACTTCTGCTC**GATAAAGTGAGATATTTGTATAAG

S3 RT-PCR analysis results for selected GRs.

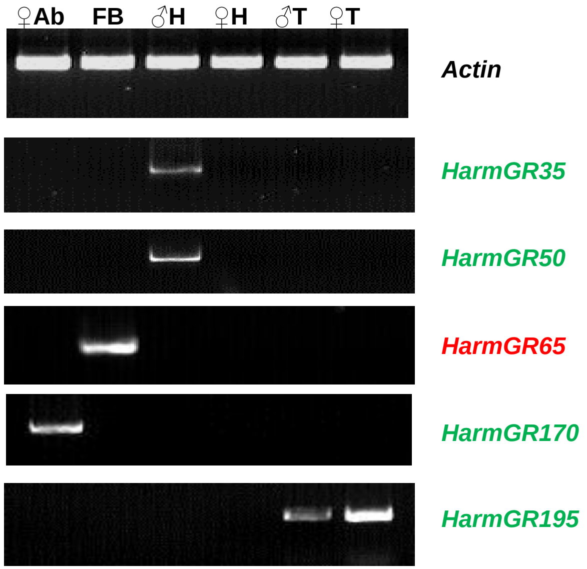

S4 3’ RACE results of HarmGR17 (Left) and 19 (Right). RED = 3’RACE primer sequences. Black bold = stop codon. Blue =poly A sequences.

ATGTTTATCCGACTTATTAGTGGATTTTATTGCAAAATAAGTTCCAATA
GCAAGATTAATTTCTTGGTGAAAACTTACTGTATATTAATCGCGACACT
AATCATTTTTTTAACAATAACTGTAGCTTTTGTTAGAGTTAACATTGAA
AGTAAATGTCACATTGGGTTTATGTCGACTCTCTACATTATAAGTGTGG
TAACTAGCATTTGTTTAAATGGGGACAACTTCGAAGATTTTTTGACGAA
AATAAGAGGAATTACCACATTACCAAATGTCGAATCCGCTGATAAAATA
ACCTTTTCAATATTTTTATTTTTCTTGTCACTGTGCTCGAGGATAGTTG
TCAATGTTAAATTTACGATTGATAACGTTCAAAGTATATCTGATCCTTT
ATTTTATGTTTCCCTGGTATCACTTACTCTTGCTACTTTACAATATTCA
GCGAGTTTTACAAGGATAATGATGTTTGAGCTTCTATGTCGCCGAATGG
TTATTCTCCGTAAACGCGTGGAAAGCGACTTGTCAATCCCAATGACATA
TCAAGCCAAAGCGATGTCGGAAGAAAAAATAAGAAGTTGTCTCCATACT
TATAAGAGTTTGTTGGACACGATCAGTGGGACTGATATGCCTATGAAGT
TTCTGGTAACTTTTTTGCTTAAAAATCTTATTGAAATTACATACCTATT
GCCATTTAATAAATATAGTTTAAT**GCGCTTTTACACTACCGGATTGTAC**
GGTCAATGTCACGCGCAATTACAAACAATTTACAAA**TGA**TGACACTTGA
AACGGAATCCAGCGAGTATAAAAACCAGGCGTATACGCGCCACAAATTC
CGTCAGTGTGAAAGCGCTCTCGTGAAGTAACGTAACGTATGTATTTGAC
CCAAAATACTTTTTACAAGTCTTATGGTTTTTTCAGACATTTATTTCAG
TCATAATATTAATGCCACAAACTTTGAACGATATATTCGGCTACGTCAT
TTTAAGAAGGAGCGTAGTAAGTACTTACTTACGTACCTTTCTTTATAAC
AGTTTTATAGTTAAACGGCTCTCTATCTCAAAAGTACCTTAAATATTT
CAAATGAAAATACTTTAGAATAGAATTTATTTCCTATAGTTCTTTTTAA
TAGAGACTTTCAATATAAATTTGTTTCTCAAATATGTTAATTTATATTT
TGCTTCATTTACAGACGAGTTATTTTATAATTTTTGCAAGTATGTTACG
TTTCGTCGTAACTTTATCACCGGCGATACTATGTGAAACGATTAACATA
AGTATAAGTAAAATCAAACTATGCATTATAAAGCAGTTACCTATGTGTG
AAGGTATATTATTTTCACATTTTATAAACTTATTTATAGGAAAAAATTG
TGGATGTTTCTATAAAAAACTTGCATTTCAGATGAAAGAACGCGTAACG
CAATACAAGACGCGATGACGTACTTGGAGCAGAACCCGTTCAAGTACAC
AATATGGCGCGTGTTCGCGGTAGACATGAGTCTGATACTGAACATGATC
GCGCTCCTCACCACATACACTGTCGCTATGGTGCAGTTCGCGCATATTT
ACGATTAATCATAATCATTATATGTGTAGTATATTTTTATTGTAAGGAG
CTTTATTACAACAGTTTGTCATGTAAAACTTCTAGCGGAATAGTAGAAT
TTTACGTTAGGAAATATTCCTTAGATTTTCTAAGCACATAGTATTG**AAA**
AAAAAAAAAAAAAAAAA

ATGGCATCTGTAAAATTAGAAGCGTTTATCAAAAGTGTTATTTTTCTTCGTCTT
TTTTGTGGATTATATTATAAACTCAGTTCAAATAAAATAACAGTTGCATTAACA
AAAATCTACTGCACGTTTGTCGCTAAAATAATTATAACTATTTTTGTTTTGTTC
ATAAAAAAATCTAAAGGTTCCTTTTCAAATTTACCTTAGTTTTGGTTTGATGTGC
TCTGGGTACCTTACGAATGTTGTTTTCAGTGCCTATTTTGATGGCGACAACTTC
ATGAAATATTTTAGCGCACTAAAAGAAATAAGAAATCCACAAGACTTCCCGTCT
TTCAGTGACATCAAGATTTCAATCATTTTATTGTTTTTTTTTGCTTCCTCAAGG
ATATTTAACTATGCGGCATACTCCGTCAGTGCCATGTTTACTTTTCAAAACCCA
TTCCCCTATAATATATATACTACTACAGCCTTTATAGGGTCAATAGCAGTTCTA
CATTCGTTGGTGAACTCGACACGAATGATGACTTTCGAATTATTGTGGCGTCGA
ATGGCCATACTCCGCAAACGCCTCGAGCAAGATCTATTAAAAGCTAGAAGATTC
GAGAACGAAGGAGACATTTTGAAAAATAATTTAAAACAATTCCTGAATACTTAC
AAAAGTATTTTGGACACGATTCGTATAAGTGAAAAACCAATGAAACTTGGGGTA
ACTGTAGTTACTAATAACAA**GTCTTCTTATTTAAATACAAATCACATTG**CAGTT
AATATTTTGAGA**TAA**GAGTTTCCAAGCGGTGCTCGATAACATCCGACATCAAAA
ATACTTCGACTTCTTTTTCCATTTCAGATACTTACGAGTTTGGTCTTATTTACT
CCTCACATGATGGATCATGCTTATACCTTCATACTCGACCAACATTCTGTTGTA
GGTATTTTGCTAAAATAGAGCTTTTATTTGATCTGATATAATAAGAAGCTATCA
CTAAAGCCTCTATGTCTTGCAGGACACGTTATTGATAATTTTAGAGGGTTCTAC
TCAATTCATTGAAACGATGTCTCCCGCTATGCTAGTGGAGATGGTCAGCTGCGA
AATTGATAGAATGAAGTTGTGTGTTACTAAGCAGTTACTCGTCTGTAAAGGTAA
CTAAATAACTAAAGATTGCATGGTATTAGTTAACAAAATTCTACGGTTGGTAAG
AAACCAGCGCGGCGTTCAAAACTAATAAGAATGTACGACAACCAGCATGCAGAA
TTTGCGGTGTGCCCATCGACAGTAAAATTTGTATTGCATACCAGAATAGAATTG
TTCCTTCAATGTATGAATCTAAAATTAAAATAAAAGGCTTCGAAAGACGATGGC
AATATGATAGAATATATTTTCAGAAAGCTGCACCCGCGACGCAATACAAGACGC
GATGACGTACTTGGAGCAGACCCCGTTCAAGTACACAATATGGGCGCGTGTTCG
CGGTAGACATGAGTCTGATACTGAACATGATCGCGCTCCTCACCACATACACTG
TCGCTATGGTACAGTTTGCGCATTTGTACAATTGATTGACCTTCAGATAGAAAT
GTCGTATTAGCACTGTAATTTATGCGTTTTTTGAGTAAAACATCTTTGAAGCAC
GGAAAAACTTCTTATTTAAATATTTTTTGGGTCTATTTCTACGAGTCGACGGAT
GCTTAACATCCATTGCTAAGTGTGTTATGGGTTTTCTAAGCATACCGAAAGAGA
CGTGTGGCTGGTATATACAGGGTGATAGGTGAATATACAGGGTTATAGGTAAAA
CACTAACAACCTTTCAGGACCATATTACTAATATCAGAAACTAAAACTTTTGTT
CTATGACTTTTAATAAGTCTCAGATTTTAGTTCATCAAAATTTTCACAC**AAAAA**
AAAAAAAAAAAAAAAAAA

S5 Comparison of lengths of the Type 1 (blue), Type 2 (red) and Type 3 (green) full-length bitter GRs of *H. armigera*.

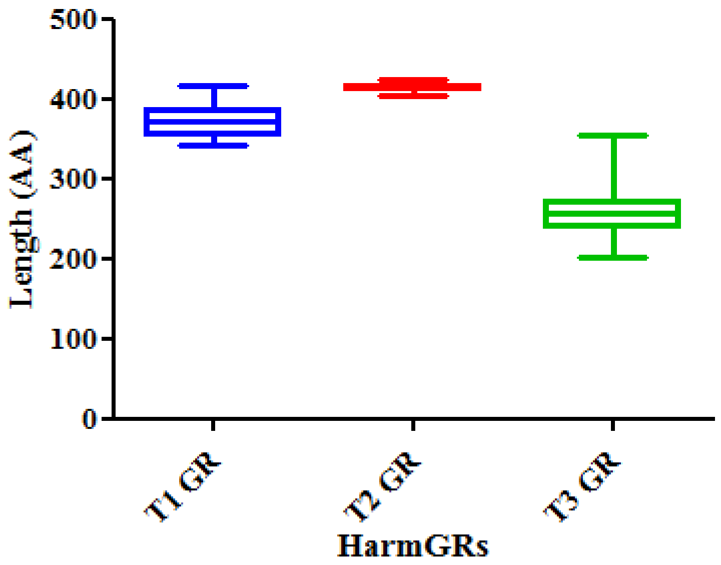

S6 Predicted transmembrane domain frequencies of Type 1, Type 2 and Type 3 bitter receptors from *H. armigera* by TMPred, HMMTOP and TMHMM.

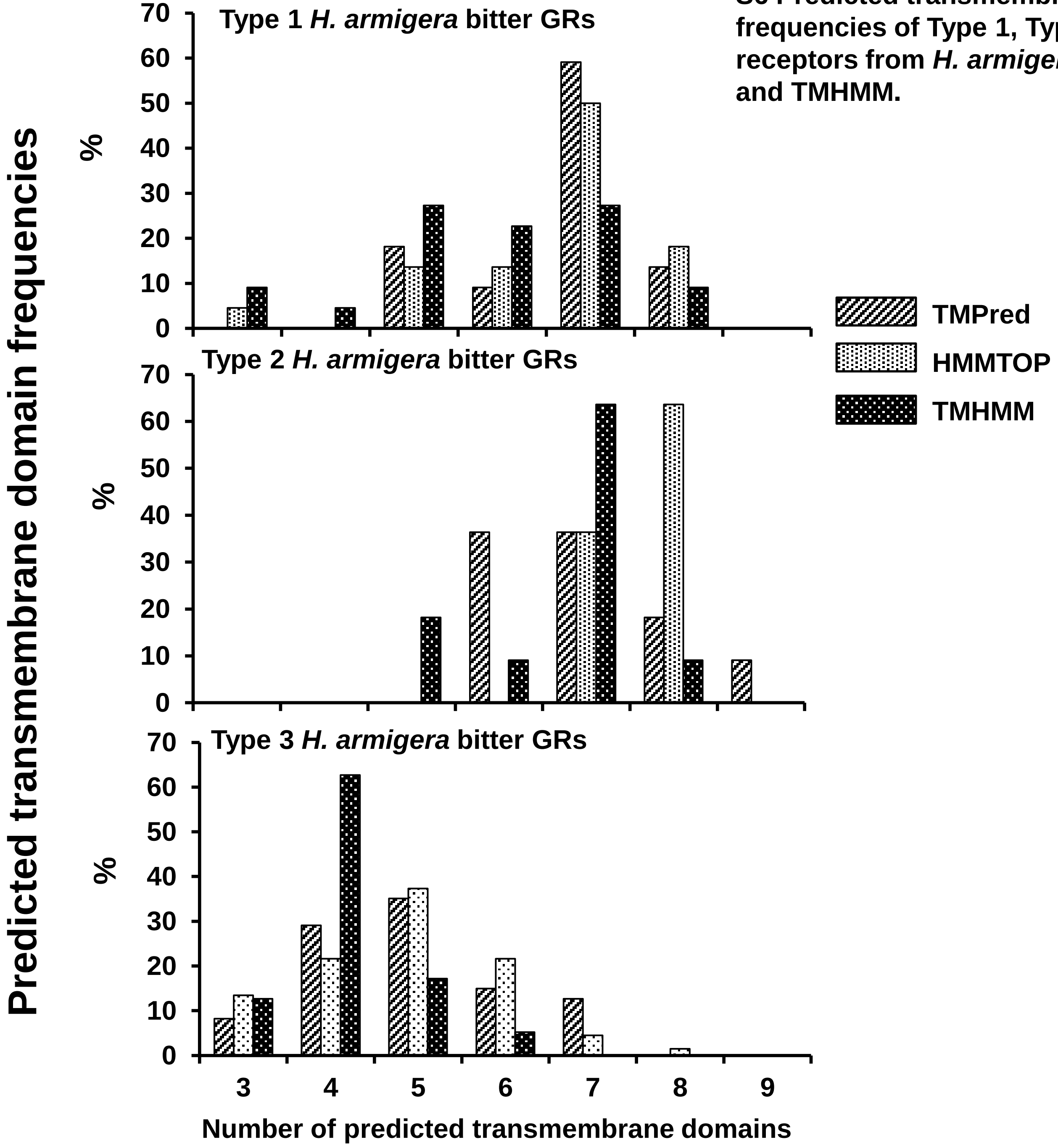

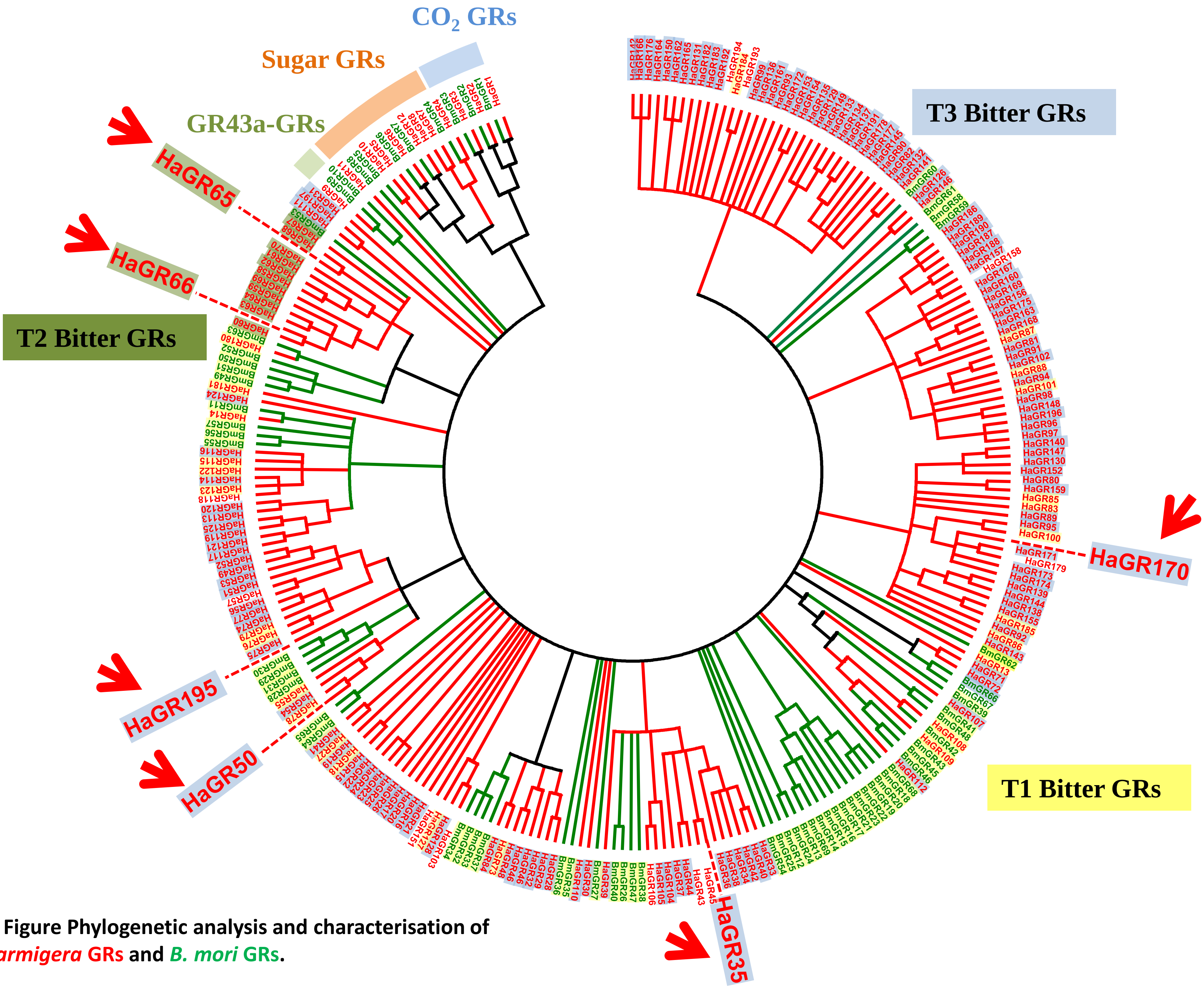

S7 Figure Phylogenetic analysis and characterisation of *H. armigera* GRs and *B. mori* GRs.
